# Development of the ICF-CY Set for Cardiac Rehabilitation After Pediatric Congenital Heart Surgery

**DOI:** 10.3389/fped.2022.790431

**Published:** 2022-01-27

**Authors:** Wen-Yi Luo, Ping Ni, Lin Chen, Qian-Qian Pan, Hao Zhang, Ya-Qing Zhang

**Affiliations:** ^1^Shanghai Jiao Tong University, School of Nursing, Shanghai, China; ^2^Shanghai Children's Medical Center, Shanghai Jiao Tong University, School of Medicine, Shanghai, China; ^3^Faculty of Education, The University of Hong Kong, Hong Kong, Hong Kong SAR, China; ^4^Editorial Department of Journal of Shanghai Jiao Tong University (Medical Science), Shanghai Jiao Tong University, Shanghai, China

**Keywords:** ICF-CY, congenital heart disease, pediatric, surgery, cardiac rehabilitation

## Abstract

**Background:**

Most children with congenital heart disease (CHD) require surgical repair, and postoperative rehabilitation is an essential step to restore the quality of life. The present study constructs and confirms the International Classification of Functioning, Disability, and Health for Children and Youth core set for children with congenital heart disease 1 year after surgery (ICF-CY-CHDS).

**Methods:**

From February 2021 to August 2021, 340 children aged 3–6 years after CHD surgery were evaluated using the ICF-CY-CHDS and analyzed using the Rasch model.

**Results:**

The final ICF-CY-CHDS contained 22 categories; it exhibited a nonsignificant χ^2^ test result for the item-trait interaction (χ^2^ = 6736.37, *p* = 0.8660, Bonferroni-adjusted *p* = 0.0023). The average severity of children was less than the average difficulty of categories (−2.26 logit <0 logit). The weighted k of all the categories was 0.964 (*p* < 0.001), and the item separation index was 0.96. The area under the ROC curve of children with a diagnosis result of heart failure was 0.866 (95% CI: 0.801 ~0.931) with good sensitivity (0.875) and specificity (0.759).

**Conclusion:**

The ICF-CY-CHDS presents a preliminary practical direction during early cardiac rehabilitation after pediatric CHD surgery, and thus provides a basis and scope for clinical evaluation and intervention program formulation.

## Introduction

The prevalence of congenital heart disease (CHD) was reported to be one per 1,000 live births worldwide between 1990 and 2017 (1). Surgery is the primary treatment for CHD. In recent years, the mortality after congenital heart surgery in pediatric heart centers has decreased to 2–3% (2). Reasons for decreasing mortality are due to the following aspects: improvements in surgical skills, the protection of the myocardium by cardiopulmonary bypass technology, advanced postoperative monitoring, and the application of extracorporeal membrane oxygenation.

However, the surviving children with critical CHD still face many problems after surgery, especially the related adverse events caused by heart failure and a decline in their quality of life. Studies have reported that the probability of heart failure in the first year after CHD surgery is high; nearly 27% of children die of heart failure 30 days after CHD surgery (3). In addition, the quality of life of these children in the first year after CHD surgery is worse (4) compared to other periods after surgery. Therefore, children need standardized outpatient follow-up, regular examinations, and physical activity guidance in the first year after CHD surgery to avoid disease-related adverse events and improve their quality of life (5). Intervening the higher risk of heart failure in the first year after CHD surgery is the key problem that needs to be solved.

The International Classification of Functioning, Disability and Health (ICF) is a global classification system developed by the World Health Organization (WHO) that focuses on health rather than diagnosis. ICF encodes the health status based on the framework of body function, body structure, activity and participation, and environmental components. The ICF version for Children and Youth (ICF-CY), published in 2007, follows the same standards (6). Currently, the ICF-CY is used to evaluate the health status of children to improve their quality of life; it is also applied for conceptual models of clinical intervention and adaptive training services (7). Previous study (8) has found readily identifiable risk factors for readmission in children after CHD surgery, including socio-demographic and clinical characteristics. Although these characteristics may not be modifiable, they are identifiable and as a result may require a comprehensive evaluation and interventions by health caregivers. The construction of the ICF-CY core sets for CHD surgery will help to extract the core issues that require high concern from clinicians. This will help physicians to identify high-risk children through core categories, to provide targeted cardiac rehabilitation.

In the present study, we constructed and investigated the ICF-CY core set in children after CHD surgery (ICF-CY-CHDS). Our study results provide a foundation to establish a cardiac rehabilitation evaluation system in the future.

## Methods

### Design

This study was a mixed study with two stages. First, the evaluation tool of cardiac rehabilitation after CHD surgery was constructed based on qualitative research, a literature review, and expert consultation. Second, the reliability and validity of the tool was clarified using quantitative research. This study was approved by the ethics committee (SCMCIRB-K2021002). This investigation conformed to the principles outlined in the Declaration of Helsinki.

### Participants

To avoid the influence of children's growth, this study evaluated preschool children (3–6 years old) within 1 year after CHD surgery with their parents' consent. For the Rasch analysis of polytomous items, Linacre (9) suggested that sample sizes of 10 per category may be sufficient. The sample size of this study was 270 (10 times 27 categories).

### Measures

#### Demographic Data

Demographic data included gender, age, the score of RACHS-1 (risk adjustment in congenital heart surgery-1) (10), follow-up time, diagnosis of heart failure, medicine taken, and parental education.

#### Heart Failure

The criteria for heart failure with a reduced ejection fraction (HFrEF) was a reduced LVEF (≤ 40%). The criteria for heart failure with a mildly reduced ejection fraction (HFmrEF) was patients with a LVEF between 41 and 49% that had mildly reduced LV systolic function. The criteria for heart failure with preserved ejection fraction (HFpEF) was (1) LVEF > 50%; and (2) objective evidence of cardiac structural and/or functional abnormalities consistent with the presence of LV diastolic dysfunction/raised LV filling pressures including raised natriuretic peptides (11).

#### ICF-CY-CHDS

**Figure 1:**
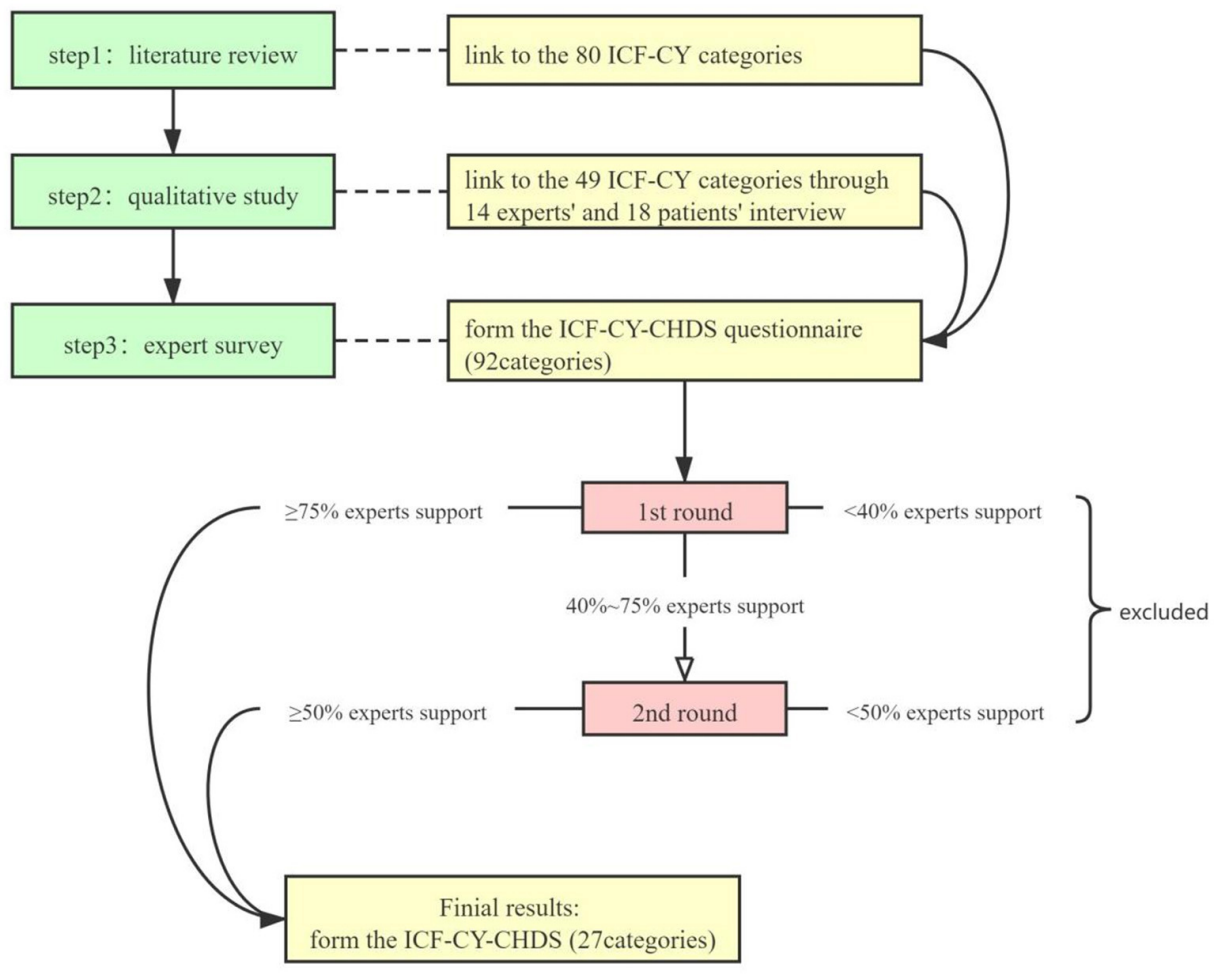
Flow chart of building the ICF-CY-CHDS.

The ICF-CY-CHDS was constructed based on the standardized three-step methodology to develop an ICF core set (see Figure 1) (12).

Step 1: Based on the MEDLINE, EMBASE, Wanfang, and CNKI databases, English and Chinese published articles with the keywords “congenital heart disease” and “cardiac rehabilitation” were searched for relevant studies from their respective inception dates to November 30, 2020. The article selection criteria were: (1) literature related to pediatric patients after CHD surgery; (2) children aged between 0 and 18 years old; (3) articles published prior to November 30, 2020; and (4) case studies, qualitative studies, animal experiments, letters, and reviews were excluded. Then, the original set of the ICF-CY category was formed based on the outcomes of the selected literature. Step 1 resulted in a total of 80 ICF-CY categories.

Step 2: Qualitative research was conducted involving semi-structured expert interviews with 14 medical staff engaged in diagnosis, treatment, follow-up, rehabilitation, and nursing related to CHD and 18 parents of children after CHD surgery in the first year. The relevant concepts in the interview content were immediately transferred to the corresponding ICF-CY category until no new concepts appeared, that is, when the interview reached saturation. After merging the literature review and qualitative research, duplicated categories were eliminated to form the ICF-CY-CHDS questionnaire. Step 2 resulted in 49 ICF-CY categories.

Step 3: Experts were invited to assess whether each category was related to the cardiac rehabilitation of children after CHD surgery. Thirty-six experts were identified from the National Pediatric Medical Center Heart Alliance and the Congenital Heart Disease Group of the Nursing Alliance. The conditions for selecting the experts were the following: (1) engaged in postoperative medical treatment, nursing, and rehabilitation regarding CHD; and (2) had >5 years of working experience. After two rounds of expert evaluations, the ICF-CY-CHDS with 27 categories was constructed.

To reduce the differences between raters in scoring the categories, four measuring guidelines were used in this study. These guidelines followed the international common ICF scoring standards from 0 to 4 (Table 1).

**Table 1:** Measuring guidelines for the ICF-CY-CHDS.

**ICF qualifiers**	**Guideline1 (%)^a^**	**Guideline 2^b^**	**Guideline 3^c^**	**Guideline 4^d^**
0	0–4	No, none, absent, negligible	No such problem over the last 1 month	0 No problem
1	5–24	Mild slide, low	Rare (<25% of the last 1 month)	1 Mild problem
2	25–49	Moderate, medium, fair	Occasional (<50% of the last 1 month)	2 Moderate problems
3	50–95	Severe, high, extreme	Frequent (more than 50% of the last 1 month)	3 Severe problems
4	96–100	Complete, total	Almost every day (more than 95% of the last 1 month)	4 Complete problems
	e.g., b530, b560	e.g., b1302, b134, b420, b430, b435, b440, b445, b510, b515, b545, d820, d880, e1101, e410, e450, e580	e.g., b122, b147, b152, d160	e.g., b280 - Wong-Baker pain scale, b410 - The New York Heart Association classification, b455 - OMNI scale, s410 - risk adjustment in congenital heart surgery-1 method, Pf1 - Family socioeconomic status

a *To transform the patient information into the percentages of the ICF calibrated scale*.

b *To transform the wording from patient reports in the ICF qualifiers*.

c *To transform the frequency with which a problem was observed during the previous month into the ICF qualifiers*.

d *To transform the scores of the selected instruments or standards into the ICF qualifiers*.

### Data Collection

From February to August 2021, data were collected in the heart center in Shanghai Children's Medical Center, China. The demographic data were collected using face-to-face interviews. Before the patients officially entered the cardiac rehabilitation process, the two nurses rated every patient using the ICF-CY-CHDS independently. All the data were recorded in electronic medical and nursing records. The researchers exported these medical data for analysis until all the data was collected. If the missing data exceeded 10%, the case was excluded.

### Data Analysis

Version 26.0 of the SPSS software suite was used to compile the descriptive statistics summarizing the patients' demographic data and conducting the critical ration (CR). CR refers to the use of the difference of the average of extreme groups to analyze the discrimination. The total scores of each subject were ranked to find the scores of the first 27% of subjects (high group) and the last 27% of subjects (low group). The mean difference was compared between the two groups in various categories and tested for significance using an independent sample *t*-test. Rasch analysis was applied using Version 3.72.3 WINSEPS. Chi-square item-trait interaction statistics were applied to evaluate the overall fit of the model for the categories of each component. A Bonferroni-corrected significance level was used to adjust for multiple comparison (*p* = 0.05/k, k = number of categories) (13, 14). The item person interaction statistics for items and persons were used to assess item fit and person fit to the model (ranging from 0.5 to 1.5). The fit of each category was indicated by the standardized residuals (z values). Z values exceeding ± 2.5 were considered to indicate a misfit to the Rasch model. Reliability was studied using the person and item separation index that ranged between 0 and 1. A value of one indicated perfect reproducibility of the person and item locations on a latent continuum. A K value of 0.81–1.00 was viewed as a near perfect agreement to evaluate the inter-rater reliability (15). Sensitivity, specificity, and area under receiver operating characteristic curve (ROC) were used to judge the predicted effect of the ICF-CY-CHDS.

## Results

### Patient Characteristics

Convenience sampling eventually yielded 340 eligible children after CHD surgery in the first year from February to August 2021. Due to the mandatory items set in the electronic record sheet, there was no missing data in the exported data. These patients had a mean age of 4.48 ± 1.05 years. Their follow-up time was 8.33 ± 2.79 months after CHD surgery. The other characteristics are shown in (Table 2).

**Table 2:** Characteristics of the study population (*n* = 340).

**Items**		**Number**	**Frequency (%)**
Gender	Male	166	48.8
	Female	174	51.2
RACHS-1	1	8	2.4
	2	230	67.6
	3	85	25.0
	4	15	4.4
	5	2	0.6
Medicine taken	Need	54	15.9
	None	284	83.5
Heart failure in the	HFrEF	1	0.3
follow-up	HFmEF	2	0.6
	HFpEF	21	6.2
	None	316	92.9
Parental education	Primary school	10	2.9
level	Junior middle school	91	26.8
	Senior middle school	64	18.8
	College and undergraduate	158	46.5
	postgraduate	17	5.0

*RACHS-1, risk adjustment in congenital heart surgery-1 method*.

*HFrEF, HF with reduced ejection fraction*.

*HFmrEF, HF with mid-range ejection fraction*.

*HFpEF, Heart failure with preserved ejection fraction*.

### Critical Ration

A percentage of 27% of the total score was used for both the high group (≥13) and low group (≤ 6). There was no statistical difference in e1101, b420, b445, and b280 according to an independent sample *t*-test (*P* > 0.05). These four categories were removed from the ICF-CY-CHDS.

### Rasch Analysis

**Figure 2:**
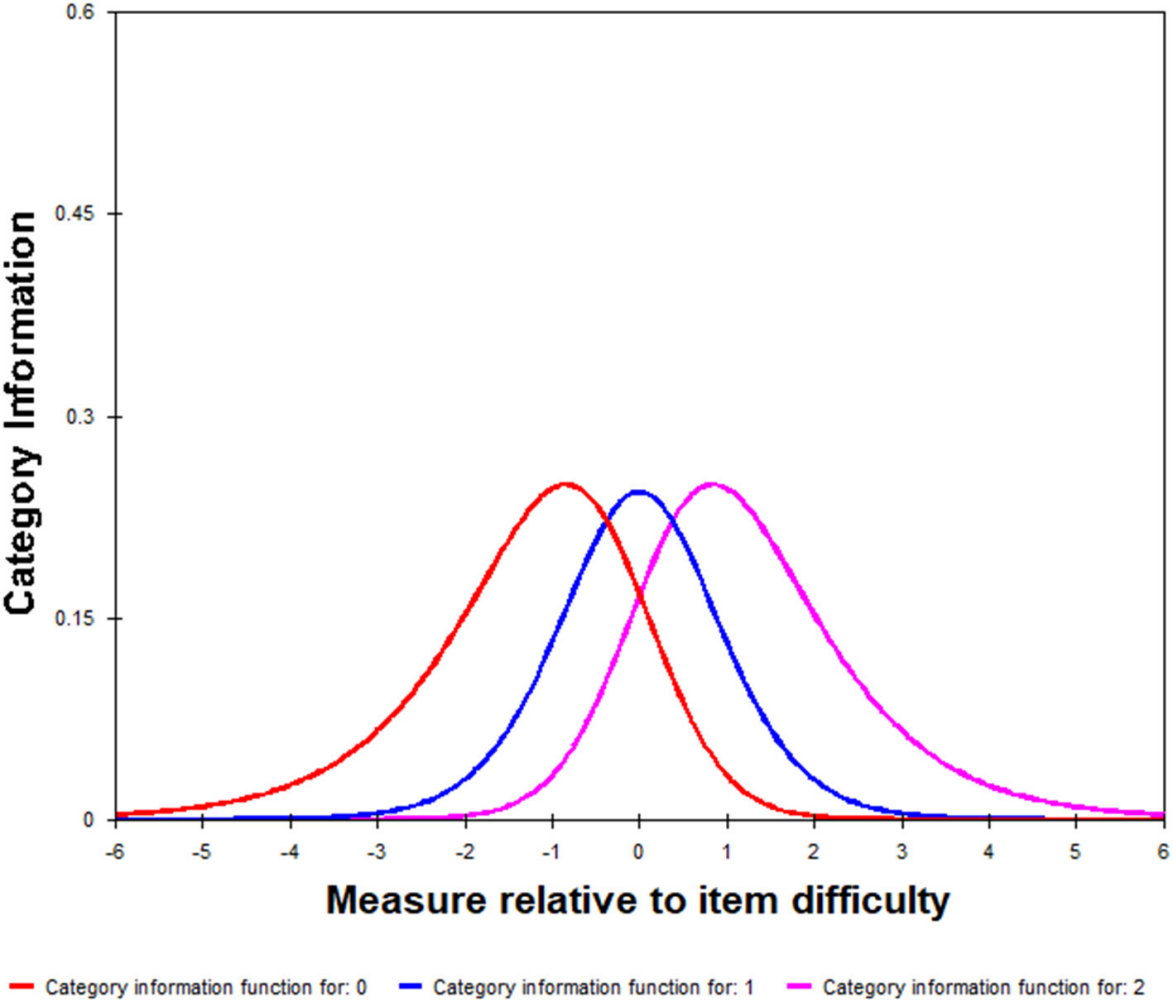
Qualifier information for the ICF-CY-CHDS.

The remaining 23 categories were entered into WINSTEPS software for the Rasch analysis, and it was found that the threshold estimate of the categories was reversed. The scoring method of all the categories needed to be adjusted using the combined strategy of different scores. Therefore, 1 and 2 were combined to be 1, and 3 and 4 were combined to be 2. Then the values of the infit and output were satisfactory (see Table 3). After the combination, the scores of various objectives increased monotonically (Figure 2).

**Table 3:** Fit of different score combinations.

**Combination strategy**	**New score**	**Number (%)**	**Infit**	**Outfit**
Combination of 1 and 2	0	6,160 (79)	0.86	0.94
scores	1	1,225 (16)	1.02	0.62
	2	302 (4)	1.06	1.35
	3	133 (2)	1.52	3.31
Combination of 3 and 4	0	6,160 (79)	0.92	0.92
scores	1	582 (7)	0.95	0.58
	2	643 (8)	0.83	0.79
	3	435 (6)	1.35	1.58
Combination of 1, 2	0	6,160 (79)	0.86	0.95
and 3 scores	1	1,527 (20)	1.03	0.70
	2	133 (2)	1.56	3.83
Combination of 1 and 2	0	6,160 (79)	0.92	0.94
scores and combination of	1	1,232 (16)	0.90	0.70
3 and 4 scores	2	428 (5)	1.28	1.47

In the first-round Rasch analysis, the 23 categories exhibited a non-significant X^2^ test result for the item-trait interaction (χ^2^ = 7438.70, *p* = 0.5574, Bonferroni-adjusted *p* = 0.05/23 = 0.0022). If the value of the item fit residuals of s410 was poor, then this category was deleted. In the second-round Rasch analysis, it was found that the infit of b530 was 2.6. After a group discussion, we decided to retain b530 because of the importance of the weight management for pediatric CHD. Finally, a total of 22 categories exhibited a non-significant χ^2^ test result for the item-trait interaction (χ^2^ = 6736.37, *p* = 0.8660, Bonferroni-adjusted *p* = 0.05/22 = 0.0023) (Tables 4, 5).

**Table 4:** Categories retained after multiple rounds of Rasch analysis.

**Item**		**Measure**	**Model S.E**.	**Infit**		**Outfit**	
				**MNSQ**	**ZSTD**	**MNSQ**	**ZSTD**
e410	Individual attitudes of immediate family members	1.96	0.38	0.93	0.0	0.75	−0.4
b510	Ingestion functions	1.61	0.31	1.46	1.3	0.60	−0.9
b440	Respiration functions	1.28	0.26	0.88	−0.3	0.81	−0.4
b147	Psychomotor functions	1.28	0.26	1.01	0.1	0.54	−1.4
b545	Water, mineral and electrolyte balance functions	1.15	0.25	1.39	1.4	1.03	0.2
b430	Hematological system functions	0.53	0.18	1.40	1.8	0.85	−0.5
b122	Global psychosocial functions	0.53	0.18	0.80	−1.0	0.70	−1.2
b134	Sleep functions	0.50	0.18	1.13	0.7	1.23	0.9
d820	School education	0.20	0.15	1.21	1.2	0.93	−0.3
d880	Engagement in play	0.12	0.15	1.00	0.1	0.71	−1.5
b515	Digestive functions	0.01	0.14	1.08	0.5	1.15	0.8
b410	Heart functions	−0.04	0.14	0.84	−1.1	0.64	−2.1
pf1	Family socioeconomic status	−0.17	0.13	0.92	−0.6	1.16	0.9
b152	Emotional functions	−0.57	0.11	0.81	−1.8	0.92	−0.5
b455	Exercise tolerance functions	−0.60	0.11	0.77	−2.3	0.79	−1.5
b1302	Appetite	−0.61	0.11	1.18	1.6	1.36	2.3
d160	Focusing attention	−0.71	0.10	0.88	−1.2	1.16	1.1
e450	Individual attitudes of health professionals	−0.84	0.10	1.13	1.4	0.97	−0.2
b560	Growth maintenance functions	−1.24	0.09	1.17	2.2	1.04	0.5
b435	Immunological system functions	−1.27	0.09	0.83	−2.5	0.97	−0.2
b530	Weight maintenance functions	−1.48	0.08	1.18	2.6	1.03	0.4
e580	Health services, systems and policies	−1.63	0.08	1.11	1.7	1.11	1.4

**Table 5:** Summary of results of the Rasch analysis.

	**Measure**	**Person (*****n*** **=** **340)**				**PSI**	**Measure**	**Item**				**ISI**	**χ^2^**	***P***
		**Infit**		**Outfit**				**Infit**		**Outfit**				
		**MNSQ**	**ZSTD**	**MNSQ**	**ZTSD**			**MNSQ**	**ZSTD**	**MNSQ**	**ZTSD**			
23 categories	−2.36	0.97	0.1	0.92	0.1	0.48	0.00	1.09	0.4	0.92	−0.4	0.97	7,438.70	0.557
Final 22 categories (delete s410)	−2.26	1.00	0.2	0.93	0.1	0.38	0.00	1.05	0.3	0.93	−0.1	0.96	6,736.37	0.866

**Figure 3:**
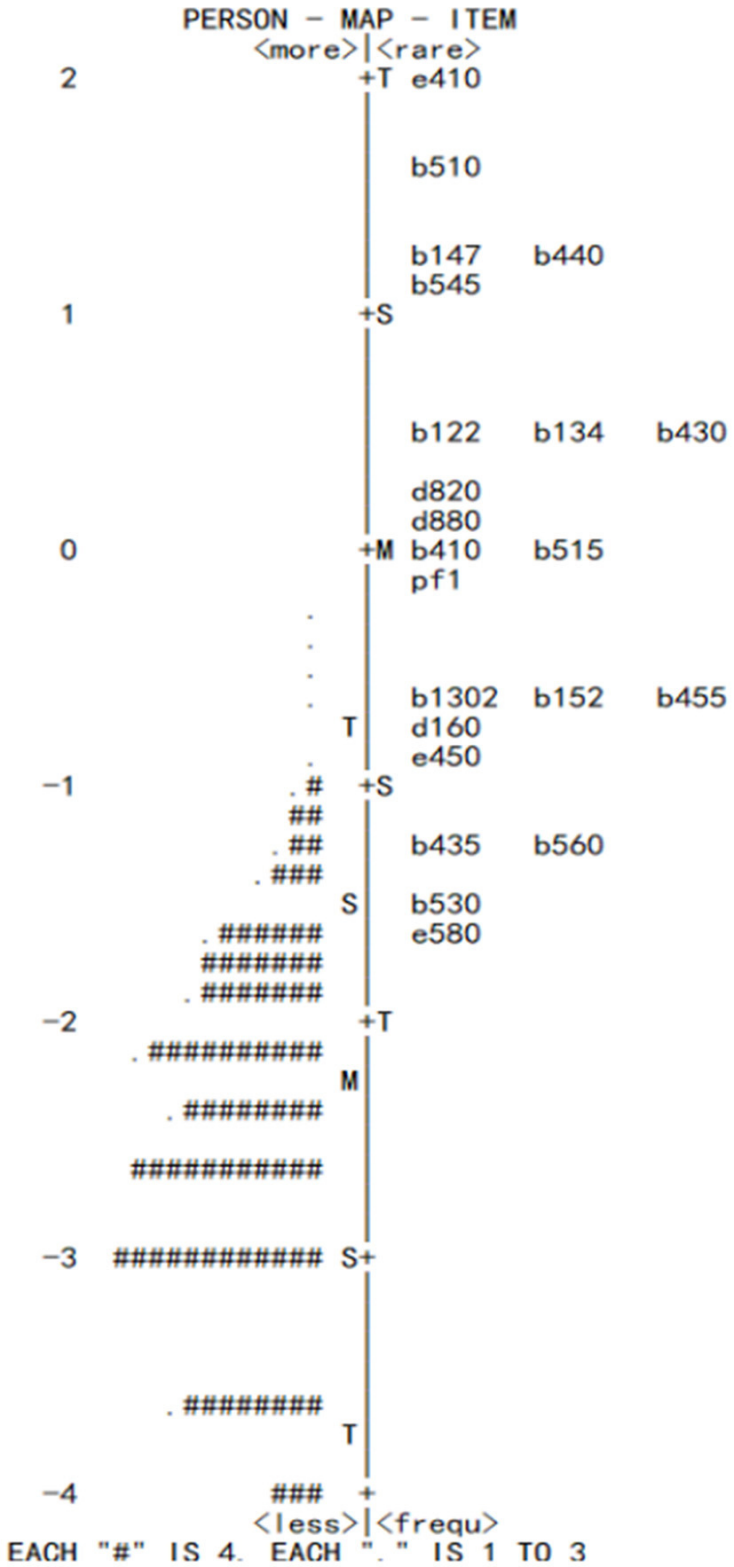
Item map of the 22-categories of the ICF-CY-CHDS for the children after CHD surgery in the first year.

The severity of children and difficulty of the categories was transformed into a logit scale to be compared directly. Figure 3 shows that the average severity of children was less than the average difficulty of categories (−2.26 logit <0 logit).

### Inter-rater Reliability of the ICF-CY-CHDS

The two nurses used the ICF-CY-CHDS to measure the 340 participants independently. The weighted k over all the categories was 0.964 (*p* < 0.001), and the weighted k of the remaining 22 categories ranged from 0.855 to 1.000 (*p* < 0.001).

### Relationship Between the Diagnosis of Heart Failure and the ICF-CY-CHDS

**Figure 4:**
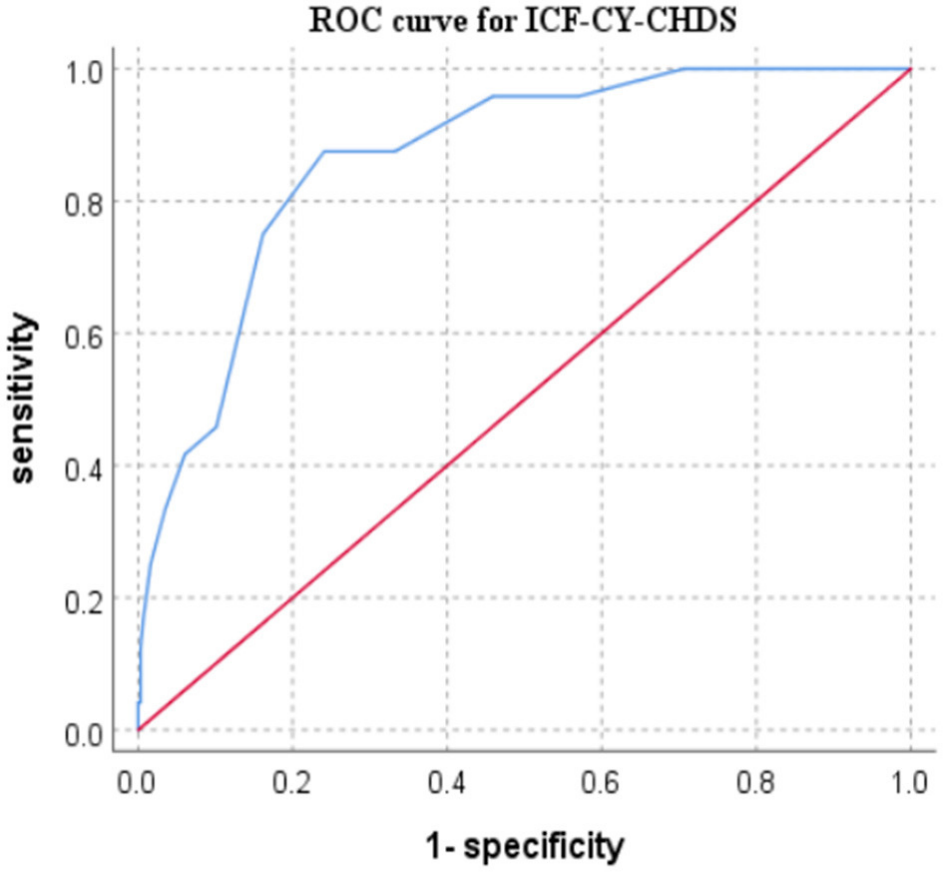
Receiver operating characteristic (ROC) curve of the ICF-CY-CHDS.

Table 6 shows that the ICF-CY-CHDS score of children with heart failure was significantly higher than that of children without heart failure (t = 8.017, *p* = 0.000). In addition, the total score was negatively correlated with the LVEF (r = −0.119, *p* = 0.029). The ROC curve with the diagnosis result of heart failure in children as the state variable diagnosed as heart failure as was shown in (Figure 4). The area under the curve was 0.866 (95% CI: 0.801–0.931), and the boundary value was seven points. The ICF-CY-CHDS had good sensitivity (0.875) and specificity (0.759) for the evaluation of heart failure in such children.

**Table 6:** Relationship between heart failure and the ICF-CY-CHDS.

	**Heart failure group (*n* = 316)**	**Non-heart failure group (*n* = 24)**	**t^a^**	***p***	**r^b^**	***p***
LVEF	69.46 ± 7.30	65.55 ± 7.94	2.516	0.012	−0.119	0.029
ICF-CY-CHDS	4.52 ± 2.93	9.63 ± 3.93	8.017	0.000		

a *Independent sample t - test*.

b *Pearson correlation analysis*.

## Discussion

In this study, it was found that 24 children (7.1%) had heart failure within 1 year after CHD surgery. The establishment of standardized cardiac rehabilitation evaluation tools in the first year after CHD surgery was conducive to the early identification and intervention of the management of high-risk children. Due to the improvement of the CHD surgery scheme, it is undeniable that most children will survive, but these children may still have cardiac insufficiency, heart failure, repeated hospitalization, and even death after CHD surgery (16). Disease-related functional problems, parental care, and the social environment may affect children's recovery after surgery (17), and the existing quality of life tools cannot fully reflect the problems of these high-risk children due to dimensional constraints. The ICF-CY-CHDS constructed in this study comprehensively identified the core issues that require clinical attention for children after CHD surgery from the biological-psychological-social model, including body function, body structure, activity and participation, environmental factors, and personal factors, all of which can comprehensively reflect the overall recovery of children during early cardiac rehabilitation.

In this study, item response theory (IRT) was used to evaluate the reliability and validity of ICF-CY-CHDS. The main reason for selecting IRT was that in addition to focusing on the total score, we paid more attention to the degree of each core category on high-risk children. The Rasch model is one of the most utilized applications of item response theory (18). Unlike classic test theory, the Rasch model assesses the internal construct validity, the optimal scoring scheme, one-dimensionality, item fit, item local independency, and item bias by subgroups of respondents. This study confirmed that the ICF-CY-CHDS fit well and was convenient for clinical medical staff to evaluate, so as to fully understand the overall rehabilitation of children. Table 5 shows that the average severity of children was less than the average difficulty of a category (−2.26 logit <0 logit), indicating that the overall level of children after CHD surgery in the first year was better than expected. Figure 3 shows that the distribution of children after CHD surgery ranged from −4 to −1, indicating that these children recovered better than we expected.

In terms of category distribution, the categories of b152, b455, b1302, d160, e450, b560, b435, b530, and e580 were below −0.5 logit. These frequent categories exposed the common problems experience by children after CHD surgery, including emotion, exercise tolerance, nutrition, susceptibility to infection, and health service system problems. E580 was located at the bottom of the category distribution, indicating this problem was common among children after CHD surgery. A recent study revealed that due to a lack of training and effective intervention methods, health care professionals were unable to provide adequate support and help for these children and their families (19). In addition, according to Chinese national data, the mortality of CHD has decreased significantly in recent years (20); however, the mortality in rural children remains higher than that of urban children. Thus, the availability of health services in rural areas, including pediatric drugs, cardiac ultrasound level, and child health services, requires further attention and resolution.

In addition, the categories larger than 1 logit involved e410, b510, b440, b147, and b545. Although these categories were rare among the children after CHD surgery, they can easily trigger serious complications in children. In this study, 7.1% of children belonged to HFpEF, and they usually had difficulty breathing (b440) and less food intake (b510), and long term dependence on medicine was needed to treat heart failure (21). Long-term chronic growth retardation in postoperative children has been confirmed to be associated with a residual shunt (OR = 35.3, *p* < 0.0001), a higher heart failure score (OR = 27.1, *p* < 0.0001), and long-term oral diuretics (OR = 20.5, *p* = 0.001) (22). Moreover, studies have revealed that complex CHD children suffer from a high chance of abnormal neurocognitive development after surgery (23), and this leads to executive function problems, social interaction obstacles, and academic difficulties (24, 25), due to the cerebral ischemia and hypoxia in different cortical areas in those children (26). In addition, the more children had postoperative disorders, the more they relied on their parents' care. Their parents always had serious psychological problems (anxiety and depression) (27) and low coping abilities (28), and this leads to the “own experience” management strategy when taking care of critical children. These family management styles can result in misjudgment and delay in disease management. Therefore, paying attention to the trend of the most difficult categories (e.g., e410, b510, b440, b147, and b545) will help to determine high-risk children.

Furthermore, this ICF-CY-CHDS not only demonstrated the level of disorder in children *via* each category, but also reflected the level of heart failure in children according to the total score. Children with an ICF-CY-CHDS over seven points were more likely to have heart failure. Therefore, the ICF-CY-CHDS can be used in the early stage of cardiac rehabilitation, and this may be helpful for clinicians to identify children with heart failure and provide personalized intervention for high-risk categories in these children.

There is one limitation of this study. The removal of some ICF-CY categories as a result of model misfit does not necessarily mean that these categories were irrelevant. They should be considered in real world settings and recorded by caregivers in the future.

## Conclusion and Implications

There is considerable variation in postoperative cardiac rehabilitation among different heart centers and different age groups in children with CHD. In addition, CHD postoperative rehabilitation generally pays too much attention to cardiopulmonary function and ignores other potential obstacles in growth and development. In the present study, for the first time, we proposed the ICF-CY-CHDS as a preliminary practice guideline and blueprint for postoperative cardiac rehabilitation in CHD children. Future studies are warranted to develop intervention schemes for different populations based on the ICF-CY-CHDS.

## Data Availability Statement

The original contributions presented in the study are included in the article/supplementary material, further inquiries can be directed to the corresponding author.

## Ethics Statement

The studies involving human participants were reviewed and Ethical approval by Shanghai Children's Medical Center, Shanghai Jiao Tong University School of Medicine (SCMCIRB-K2021002). Written informed consent to participate in this study was provided by the participants' legal guardian/next of kin.

## Author Contributions

W-YL and Y-QZ: conceptualized, designed the study, and drafted the initial manuscript. HZ: designed the data collection instruments and coordinated and supervised data collection. PN and LC: conducted the data collection and reviewed and revised the manuscript. Q-QP: conducted the Rasch analysis. All authors approved the final manuscript as submitted and agree to be accountable for all aspects of the work.

## Funding

This work was supported by the scientific research project from the Shanghai Municipal Health Commission (20194Y0479) and Shanghai Jiao Tong University School of Medicine: Nursing Development Program that belongs to W-YL.

## Conflict of Interest

The authors declare that the research was conducted in the absence of any commercial or financial relationships that could be construed as a potential conflict of interest.

## Publisher's Note

All claims expressed in this article are solely those of the authors and do not necessarily represent those of their affiliated organizations, or those of the publisher, the editors and the reviewers. Any product that may be evaluated in this article, or claim that may be made by its manufacturer, is not guaranteed or endorsed by the publisher.
